# Nephroblastoma - A 25-year review of a South African unit

**Published:** 2014-09-25

**Authors:** YT Visser, R Uys, A van Zyl, DC Stefan

**Affiliations:** * Department of Paediatrics and Child Health, Stellenbosch University, Faculty of Health Sciences, Tygerberg Hospital

**Keywords:** SIOP = Société International D’Oncologie Pédiatrique Protocol, NWTS = National Wilms’ Tumor Study Protocol

## Abstract

Abstract

Rationale: To determine the outcome of patients with nephroblastoma in a South African hospital. Objective: To determine if there is a difference in the outcome of patients with nephroblastoma comparing two treatment protocols SIOP (Société International D’Oncologie Pédiatrique Protocol) versus NWTS (National Wilms’ Tumour Study Protocol).

Methods and results: A retrospective audit of 25 years (1983-2007), of children diagnosed with nephroblastoma in Tygerberg Hospital. One hundred and seven patients were included in the study and 98 were analyzed. The average age at diagnosis was 3.8 years. Most patients (37%) presented with stage 1 of the disease, followed by patients with stage 3 (27%). Most patients were treated according to the SIOP protocol (61%). Gender and race did not influence the outcome. Patients with stage 1 and 2 of the disease had the best outcome (76% versus 43% for stages 3 and 4). The SIOP group had a better outcome than the NWTS group (p value 0.001). The two groups had an equal distribution of the stage of presentation. The tumor volumes were bigger in the NWTS group (1004cm3 compared to 613cm3). Nutritional status did not influence the outcome although more patients were underweight for age in the SIOP group. The statistical methods used were: Kaplan Meier, Gehan’s Wilcoxon Test, Chi –square test and the Fisher exact test.

Discussion:Contrary to the other studies, patients treated according to the SIOP protocol had a statistically significant better outcome. Larger collaborative studies are needed to investigate this result in Africa.

Abbreviations: SIOP = Société International D’Oncologie Pédiatrique Protocol, NWTS = National Wilms’ Tumor Study Protocol

## Introduction

Childhood cancers are rare, comprising about 1% of all cancers. Nephroblastoma or Wilms tumor (WT) accounting for 6-7% of all childhood cancers [**1**] and is the most common renal tumour in childhood. In 2010, data from the tumour registry of the South African Children’s Cancer Study Group (SACCSG) showed that WT was the fourth most common childhood cancer in South Africa [**2**]. The major improvements in the diagnosis and treatment of childhood cancers over the last 50 years have resulted in a high cure rate of approximately 90% in developed countries [**1**]. Sadly, the success rate is far lower in South Africa and other developing countries, mostly due to a delay in seeking medical attention or to a lack of access to the health care [**1**] or due to the optimal therapy not being available [**3**]. 

 Race disparities have been studied in the past; in 2011, a study by Jason et al found that African people had a higher incidence of WT than Caucasians. African patients were 79% more likely to develop WT than Caucasian patients and they tended to present with a more advanced disease, but the overall survival rate was the same [**4**].

 Treatment modalities have been extensively studied over the past 25 years. The National Wilms Tumour Study Protocol (NWTS) and the Société International D’Oncologie Pédiatrique Protocol (SIOP) are currently the 2 most common protocols, being used globally. The NWTS protocol stipulates surgery upfront, followed by neo-adjuvant chemotherapy, while the SIOP protocol begins with chemotherapy after which surgery is performed. The management of Wilm’s tumor was previously researched in the developing countries (India) comparing the two protocols and showing similar equivalent clinical outcomes [**5**]. 

 The NWTS protocol was initially used (from 1983) in Tygerberg hospital in Cape Town, South Africa, but, in 1989, it was decided that the SIOP protocol should be changed. The rationale for using a different protocol was based on the clinical presentations of the children: large tumors at diagnosis, advanced disease and plans for a common national protocol. The pre-operative chemotherapy was utilized to decrease the tumor volume in order to make surgery easier and to reduce the complication rate thereof.

## Methods

The study population included all paediatric patients (<15 years) diagnosed and treated for nephroblastoma in Tygerberg Hospital’s Pediatric Oncology unit from 1 January 1983 to 31 December 2007.

 Data were collected from the Tygerberg Hospital tumor registry and from the folders of the patients.

 The following information was captured for each patient: date of birth, date of diagnosis, gender, ethnic group, staging, treatment protocol, surgery, pathology and radiotherapy reports, nutrition and outcome. 

 All the diagnosed cases were histologically confirmed by a pathologist and reviewed by a tumor board. 

 The extracted data was recorded in Excel format. Descriptive statistics including frequency tables, histograms, means and standard deviations was performed. Data was compared by using Chi square or test calculations and ANOVA or cross tabulations depending on the type of data. The other methods used were: Kaplan Meier curves, Gehan’s Wilcoxon Test, and the Chi–square test.

 Ethical Approval

 The ethical approval for this study was obtained from the Health Research Ethics Committee of the University of Stellenbosch. The superintendent of Tygerberg Hospital approved the retrieval of the data from the Tygerberg Hospital folders. Confidentiality of the patients was maintained at all times. Individual consent was not required as it was a descriptive retrospective study.

## Results

Location

 Tygerberg Hospital is a tertiary hospital located in Parow, Cape Town, serving the Eastern Metropolitan region of Cape Town and the North-Eastern districts of the Western Cape Province. The hospital was officially opened in 1976 and provides healthcare to over 3.6 million people (2.4 million children), being the largest hospital in the Western Cape and the second largest hospital in South Africa.

 The patients in this study came from the Tygerberg Hospital’s drainage area (Western Cape 61%), followed by Namibia, Northern Cape and Eastern Cape provinces. 

 Ethnicity

 South Africa consists of a diverse population in terms of ethnicity, but this can broadly be classified into Black, White or Caucasian and coloured or Mixed ethnicity. The Coloured population genetically consists of an ancestral mix between European and various Southern African Black tribes. This ethnic group is widespread across South Africa but there is a higher concentration of this population group specifically in the Western Cape region. The percentage distribution of ethnic groups shows 32.9% blacks, 48.8% coloured, 15.7% whites, and 1% Asian/Indian [**6**]. 

 Demographic results

 One hundred and seven patients in Tygerberg Hospital were diagnosed with nephroblastoma between 1 January 1983 and 31 December 2007. Ninety eight patients were included in the study; 9 patients were excluded due to a lack of complete information (including stage V disease). There were 54 girls and 44 boys. The average age at diagnosis was 3.8 years with most patients being coloured* (52%), followed by black (34%) and white patients (14%). The colored females (35%) were the most numerous.

 Staging and diagnosis

 All the patients were staged according to the international guidelines. 

 The staging of nephroblastoma was based on abdominal ultrasound, chest radiography, abdominal computerized tomography (CT scan) of abdomen and, if indicated, the chest. The staging was completed after surgery with a pathology report.

 The survival rate was correlated with the stage of the disease and the ethnic group. 

 Stage 1 of the disease was most common at presentation (37%), followed by stage 3 (24%) and stage 4 (21%) of the disease for the whole group.

 Pathology data from only 38 patients was analyzed. The average tumor volume in the SIOP was considerably smaller (613cm3 ) than in the NWTS group (1004cm3) due to the preoperative chemotherapy effect.

 Protocols and outcome 

 The patients were treated according to the NWTS protocol, from 1983-1989 and according to the SIOP protocol from 1989 onwards. 

 There were 59 cases (60%) in the first group and 39 cases (40%) in the second group.

 There were 50 patients who received a curative radiotherapy (an average of 27 GY over 3-4 weeks, according to the protocol).

 The overall survival for the whole group was of 76.5%, irrespective of the treatment protocol used. There was no statistically significant difference between male and female (75% of males and 78% of females).

**Fig. 1:**
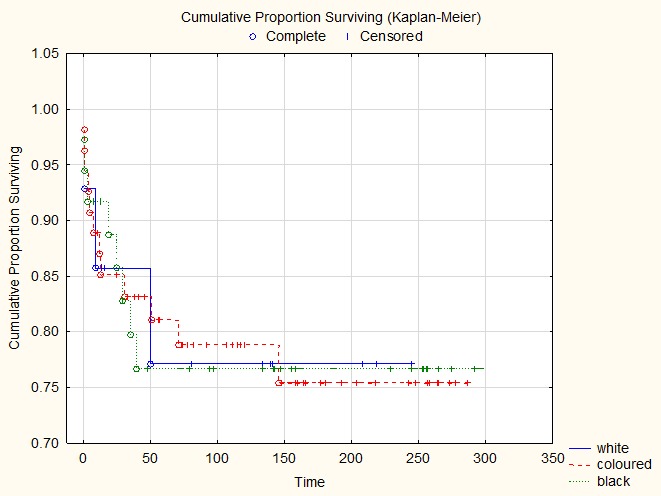
Correlation of survival with the ethnic group

 The overall survival between different ethnic groups was similar: 76.5% for the coloured population, 78.6% for the whites and 75.8% for the black patients (**Fig. 1**).

**Fig. 2:**
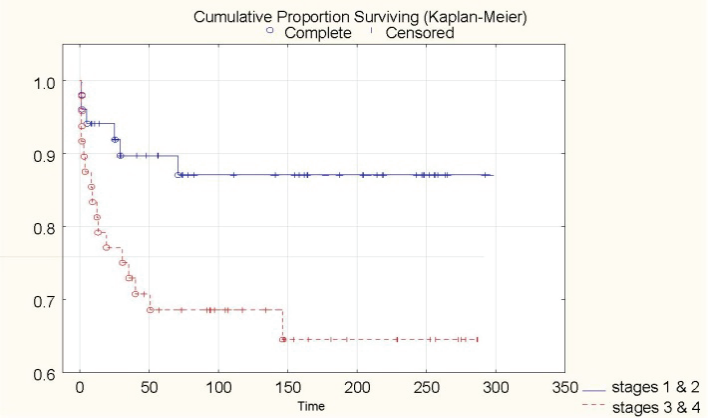
Correlation of stages and outcome (p-value=0.01)

 The outcome of patients with stage 1 and 2 approaches the values reported in developed countries: 81.6% and 93.3%. Less rewarding results were obtained after the treatment of stage 3 and 4, respectively 75% and only 57% for the advanced, metastatic disease (**Fig. 2**). Patients with stage V were not analyzed due to the lack of information.

 The overall survival rate for early stages (1 and 2) varied between 80% for whites, 82% for the colored population and 88% for the black children. The outcome for stages 3 and 4 for the blacks was 69%, for the colored group 64% and 75% for the whites. 

 Comparing the groups treated with the 2 protocols, the distribution of stages was similar: both groups had 54% of the patients with stage 1 and 2 disease and 46% of the patients with stage 3 and 4 disease. 

**Fig. 3:**
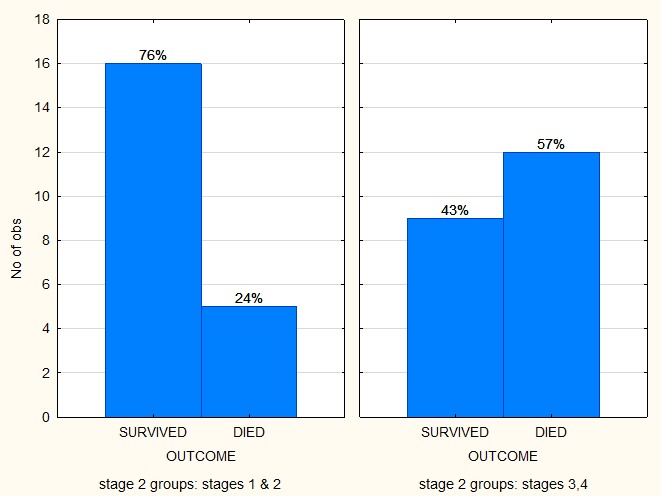
Comparing the outcomes of the stages treated with the NWTS protocol

**Fig. 4:**
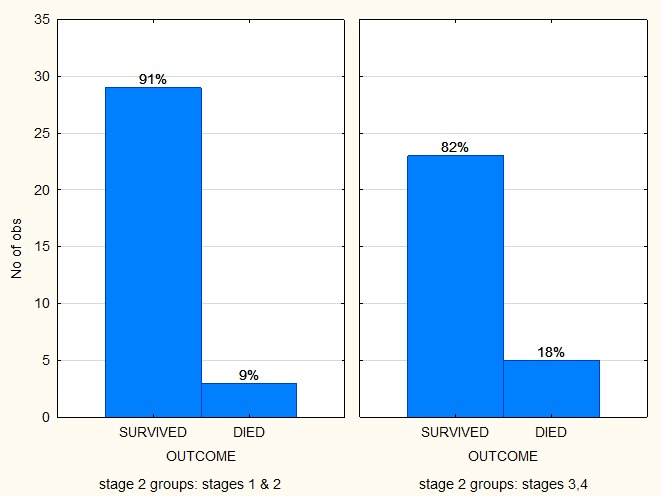
Comparing the outcomes of the stages treated with the SIOP protocol

 Comparing the outcome between the 2 groups, the survival for all stages in NWTS was 61.5% and 86.4% for SIOP with a marked difference between the stages (**Fig. 3,Fig. 4**). 

 The analysis of the nutritional status showed that 7.5% of the patients in the SIOP group were underweight for age compared to 2.8% in the NWTS group (underweight for age = weight between -2 and -3 Z score). The weights of 12 patients in the SIOP group and 2 patients in the NWTS group were unavailable. 

## Discussion

 Nephroblastoma is a common childhood tumor in Africa [**7**] and has, in developed countries a good prognosis if treated early. 

 There are epidemiological particularities regarding the disease, presentation and response to treatment in Africa.

 There are no published studies to compare the 2 common protocols used for the treatment of the African child with Wilms tumor.

 The overall survival approaches 90% in developed countries. For a variety of reasons, the survival rates seldom approach these figures in the developing countries [**8,9**]. 

 There were slightly more female than male patients in our study and this is not typically of previous reported studies [**8,9**], although the other reports have shown that there is a varying male to female ratio that ranges from 2:3 to 1:1 [**10,11**]. 

 The median age of children with nephroblastoma is reported to be of 42 months of age [**8**], which is the same as our median age of 44 months. A similar South African study showed a median age of 39 months [**11**]. There is a study from the GFAOP group which reported a median age of only 36 months in its cohort of patients [**3**]. 

 The racial distribution of our patients reflects the population of South Africa and is representative of Western Cape. It is stated in the literature that nephroblastoma is more common in Africa than in the developed countries [**7**]. 

 Stage, with histology, is one of the two most important predictors of prognosis and survival in children with nephroblastoma [**12**]. 

 This study did not gather data on the histology of the operative specimens but data on the staging was obtained. Stage 1 of the disease was most common at presentation (37%), followed by stage 3 (24%) and stage 4(21%) of the disease for the whole group. This differs from other figures from the other African countries which quote that over 70% of their patients had an advanced stage at presentation [**10**]. 

 The study of the GFAOP [**3**] showed an incidence of stage 1 of the disease at 34% while advanced stage disease only accounted for 40% of their patients.

 The overall survival approaches 90% in developed countries. For a variety of reasons, the survival rates in the developing countries seldom approach these figures [**8,9**].

 The two different treatment strategies, SIOP and NWTS, which are widely accepted and used internationally showed previously similar outcome results [**5**]. 

 The group treated according to the SIOP protocol in this study had a significantly better outcome than the NWTS patient group, especially for the patients with stages 1 and 2 of the disease (91% versus 76%). Patients with stage 3 and 4 of the disease in the SIOP group had a survival rate of 82% versus 43% for the NWTS group. The SIOP group had a better outcome than the NWTS group (p value 0.001).

 Most of our patients were treated according to the SIOP protocol (61%). Gender and race did not influence the outcome which is described for the first time in literature for an African population. A study from Poole [**13**] showed that their stage 4 survival rate by using SIOP was of approximately of about 45%, while another South African study [**11**] using NWTS had a survival rate of 54%.

 Our two groups analyzed had an equal distribution of stage of presentation. 

 The average tumor volume in the NWTS group was significantly larger than in the SIOP group (1004cm3 compared to 613cm3) since the SIOP group received pre-operative chemotherapy. Surgical complications with tumor spillage did not contribute to the upstaging of the tumor and there was no significant change in the surgical methods during the study time period.

 The nutritional status did not influence the outcome although more patients were underweight for age in the SIOP group. There were no major demographic differences between the two patient groups. On the analysis of the patients’ nutritional status, it was found that they were mostly malnourished with more patients being underweight in the SIOP group, but that did not have any effect on the outcome.

 Not enough information was available to correlate the pathology risk protocol with the outcome. Pathology reporting has changed and has become more sophisticated over the years and thus could have had an effect on the results. Anaplasia which often leads to chemotherapy resistance was identified in only 2 patients, one in each group. 

 Protocols were correctly followed and analyzed in all cases and there was no evidence to suggest a deviation from the protocols used. All patients had chemo- and radiotherapy as per protocol. Supportive care could have played a role in the improved outcome in more recent years when the SIOP protocol was used, but this is difficult to quantify. There were no major changes in the supportive care provided to the two patient groups. 

 This study confirms that gender and race did not influence the patient outcome. The statistical data shows that the main variable affecting the outcome is the stage of presentation and the protocol used. The only factor that remained a significant predictor of death was the treatment protocol used factor which was previously not described. 

 Possible reasons for the difference in results in comparison to the literature include differences in demographic and genetic characteristics, nutritional status, tumor volume, and pathology risk reporting protocols in the different eras, improvement in surgical skills, as well as protocol deviation and supportive care. 

 No genetic testing was available at Tygerberg hospital during the study period, thus this could not be investigated further.

 Limitations of the study

 There are a number of limitations to the study. The data only represents children seen at Tygerberg Hospital pediatric oncology unit. This was a small retrospective study representing a specific population. Some patients were not included in this study since essential information was not available. With regards to the pathology reports, since reporting methods also changed during the study period, it complicated the interpretation of the tumor volumes. In addition, only half of the patients’ tumor volumes could be found. 

 Conclusions

 The result that our patient population has a better outcome when treated with the SIOP protocol is highly significant and needs further research. This study also demonstrates that the differences in the ethnic group were not significant in the outcome, black, colored and white children having similar survival graphs when presented with the same stages.

 Larger studies are needed to identify the optimal treatment protocol for the treatment of the African children with nephroblastoma and a better characterization of the disease.

 Acknowledgements

 The authors wish to thank Mrs. Rina Nortje, data manager of Tygerberg hospital tumor registry, for the capturing of and assistance in data provision. They would also like to thank Dr. Martin Kidd for the statistical data provision.
